# The structural, morphological and thermal properties of grafted pH-sensitive interpenetrating highly porous polymeric composites of sodium alginate/acrylic acid copolymers for controlled delivery of diclofenac potassium

**DOI:** 10.1080/15685551.2016.1259834

**Published:** 2016-11-21

**Authors:** Aamir Jalil, Samiullah Khan, Fahad Naeem, Malik Suleman Haider, Shoaib Sarwar, Amna Riaz, Nazar Muhammad Ranjha

**Affiliations:** ^a^ Faculty of Pharmacy, Bahauddin Zakariya University, Multan, Pakistan; ^b^ Department of Pharmacy, COMSATS Institute of Information Technology, Abbottabad, Pakistan

**Keywords:** Highly porous polymeric composites, diclofenac potassium, thermal and morphological properties, *in vitro* release, structural properties

## Abstract

In present investigation new formulations of Sodium Alginate/Acrylic acid hydrogels with high porous structure were synthesized by free radical polymerization technique for the controlled drug delivery of analgesic agent to colon. Many structural parameters like molecular weight between crosslinks (*M*_*c*_), crosslink density (*M*_*r*_), volume interaction parameter (*v*_2,*s*_), Flory Huggins water interaction parameter and diffusion coefficient (*Q*) were calculated. Water uptake studies was conducted in different USP phosphate buffer solutions. All samples showed higher swelling ratio with increasing pH values because of ionization of carboxylic groups at higher pH values. Porosity and gel fraction of all the samples were calculated. New selected samples were loaded with the model drug (diclofenac potassium).The amount of drug loaded and released was determined and it was found that all the samples showed higher release of drug at higher pH values. Release of diclofenac potassium was found to be dependent on the ratio of sodium alginate/acrylic acid, EGDMA and pH of the medium. Experimental data was fitted to various model equations and corresponding parameters were calculated to study the release mechanism. The Structural, Morphological and Thermal Properties of interpenetrating hydrogels were studied by FTIR, XRD, DSC, and SEM.

## Introduction

Drug delivery systems (DDS) improve the administration and efficacy of pharmaceutical compounds including antibodies, peptides, vaccines, drugs and enzymes. Numerous methods have been adopted for successful delivery of biologicals and pharmaceuticals to systemic circulation such as oral pills, injections, capsules etc. Pharmaceutical and biological therapeutics are often limited by short half-lives, poor bioavailability, and physical and chemical instability. Physical instability mainly includes alteration of highly ordered protein structure, leading to undesirable processes such as denaturation, aggregation and precipitation. Reactions such as oxidation, deamidation, hydrolysis and racemisation contribute to the chemical instability of drugs. These limitations have given rise to substantial research focused on the development of novel DDS.[1]

Hydrogels are hydrophilic .mixture having the attributes of both the solid and liquids. Hydrogels refers to three dimensional (3-D) cross-linked polymeric network with the ability of imbibing large amount of water or biological fluids within their porous structure.[2] Hydrogel are sometime established as colloidal gel in which aqueous phase is a dispersion medium.[3] Chemical initiation, ionizing radiation initiation, semi interpenetrating polymerization and inter polymeric complexes forms the network structures of the hydrogels.[4,5] Physical or chemical crosslinking helps to maintain the firm three dimensional structures of the hydrogels.[6] Hydrogels have been extensively used as drug delivery devices, contact lenses, catheters, wound dressings and biodetector in the pharmaceutical field.[7,8] Hydrogels are also termed as ‘intelligent gels’ or ‘smart gels’ receiving, transmitting and resulting in a useful response is termed as the smartness ability of the material.[9] Because of the smartness of the hydrogel they have the ability to respond to different stimuli i.e. pH, temperature, chemical environment, ionic strength, enzymes or the presence of specific ligands etc.[10]

Acrylic acid, a synthetic monomer is pH sensitive because of the presence of enormous no of carboxylic acid groups and therefore enormously utilized in the site specific drug delivery devices particularly to colon.[11] Acrylic acid is a principle pH responsive polyelectrolyte and commercially available super absorbent therefore pH responsive polymeric matrix of acrylic acid have been researched comprehensively.[12] pH responsive, electro responsive & thermo responsive attributes have been elaborated in interpenetrating and copolymer formulations of the acrylic acid.[13] The carboxylic acid group of the acrylic acid has high tendency to ionize; interact strongly with the aqueous phase molecules and show simultaneous change in swelling pattern to pH variations and ionic strength.[14] Acrylic acid has peak value ranges from 4.5 to 5.0.[15] At the pH of 7.4 acrylic acid gives significant swelling due to ionization of an ionic carboxylic group.[16]

The incorporation of the natural polysaccharides in novel drug delivery devices eventually becomes a subject of passionate research since for their biocompatibility and biodegradability.[17] Polysaccharides based natural polymers gained the attention of the researchers towards the development of natural polymer based hydrogels.[18] Sodium alginate is the sodium salt of alginic acid, a natural polymer mainly obtained from brown algae.[19] Sodium alginate is linear, non-branched anionic polymer of polysaccharide family having repeating units of alpha-glucoronic acid & 1, 4-linked B-D-mannuronic acid.[20] Sodium alginate is extensively employed as additive in food, cosmetics, pharmaceutical industry due to its gelling capability, bracing properties and highly viscous behavior in aqueous solution.[21,22] Sodium alginate has been extensively utilized as support for controlled drug delivery due to its faster degradation and bio compatibility.[23]

**Figure 1.**
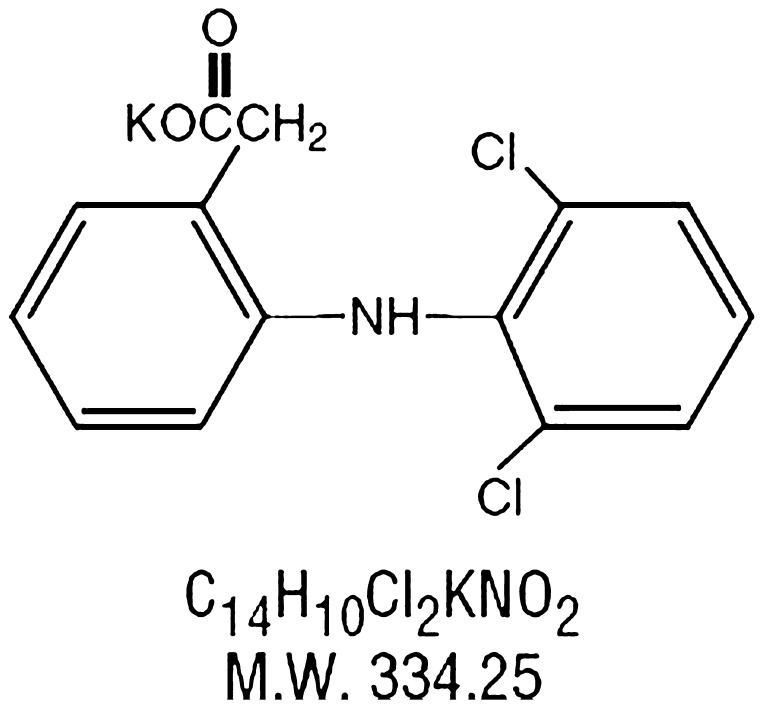
Molecular structure of diclofenac potassium.

Diclofenac potassium (C_14_H_10_Cl_2_NKO_2_) is a synthetic phenylacetic acid derivative having analgesic, antipyretic and anti-inflammatory action with plasma half-life of 1–2 h. The primary mechanism responsible for its anti-inflammatory, antipyretic, and analgesic action is thought to be inhibition of prostaglandin synthesis by inhibition of cyclooxygenase (COX). It also appears to exhibit bacteriostatic activity by inhibiting bacterial DNA synthesis. Inhibition of COX also decreases prostaglandins in the epithelium of the stomach, making it more sensitive to corrosion by gastric acid. Diclofenac has a low to moderate preference to block the COX2-isoenzyme (approximately 10-fold) and is said to have, therefore, a somewhat lower incidence of gastrointestinal complaints than noted with indomethacin and aspirin. Figure 1 indicates the structure of diclofenac potassium.

In the present work novel highly porous hydrogels based on Sodium Alginate/Acrylic acid (NaAlg/AA) copolymers are synthesized by free radical polymerization technique. These novel polymeric matrices have developed with intentions of applications in the controlled delivery of diclofenac potassium to colon. The prepared hydrogel samples were used to answer the following questions; (1) To develop different samples with different composition and degree of crosslinking. (2) To investigate the effect of composition and crosslinking ratio on dynamic and equilibrium swelling study in phosphate buffer solutions of variable pH values. (3) To investigate the effect of pH and composition on release of model drug in phosphate buffer solutions of variable pH values and to confirm the controlled delivery of model drug to colon. (4) To evaluate sol–gel fraction analysis, porosity measurement, molecular weight between the crosslinks and diffusion coefficient. (5) To evaluate the best release mechanism by applying various mathematical release models. (6) Characterization of hydrogel samples were carried out by using various characterization tools like Fourier transform infrared (FTIR) spectroscopy, X-ray diffraction (XRD), Differential scanning calorimetry (DSC) and scanning electron microscopy (SEM) to determine the network formation, crystallinity and morphology of the hydrogel samples respectively.

## Experimental

### Material

For the preparation of highly porous hydrogels, Acrylic acid (AA) (Mw ∼ 72.06 g mol^−1^) (Purity 98%, Sigma Aldrich) was used as monomer, and sodium alginate (Purity 98.7%) (Shanghai chemicals) was used as polymer. Ethylene Glycol Dimethacrylate (EGDMA, Sigma Aldrich) and ammonium persulphate (APS) (Merck) was used as crosslinking agent, and as an initiator respectively. All other reagents employed in the experimental work are of analytical grade and were used as received.

### Preparation of hybrid polymeric network of NaAlg/AA

**Figure 2.**
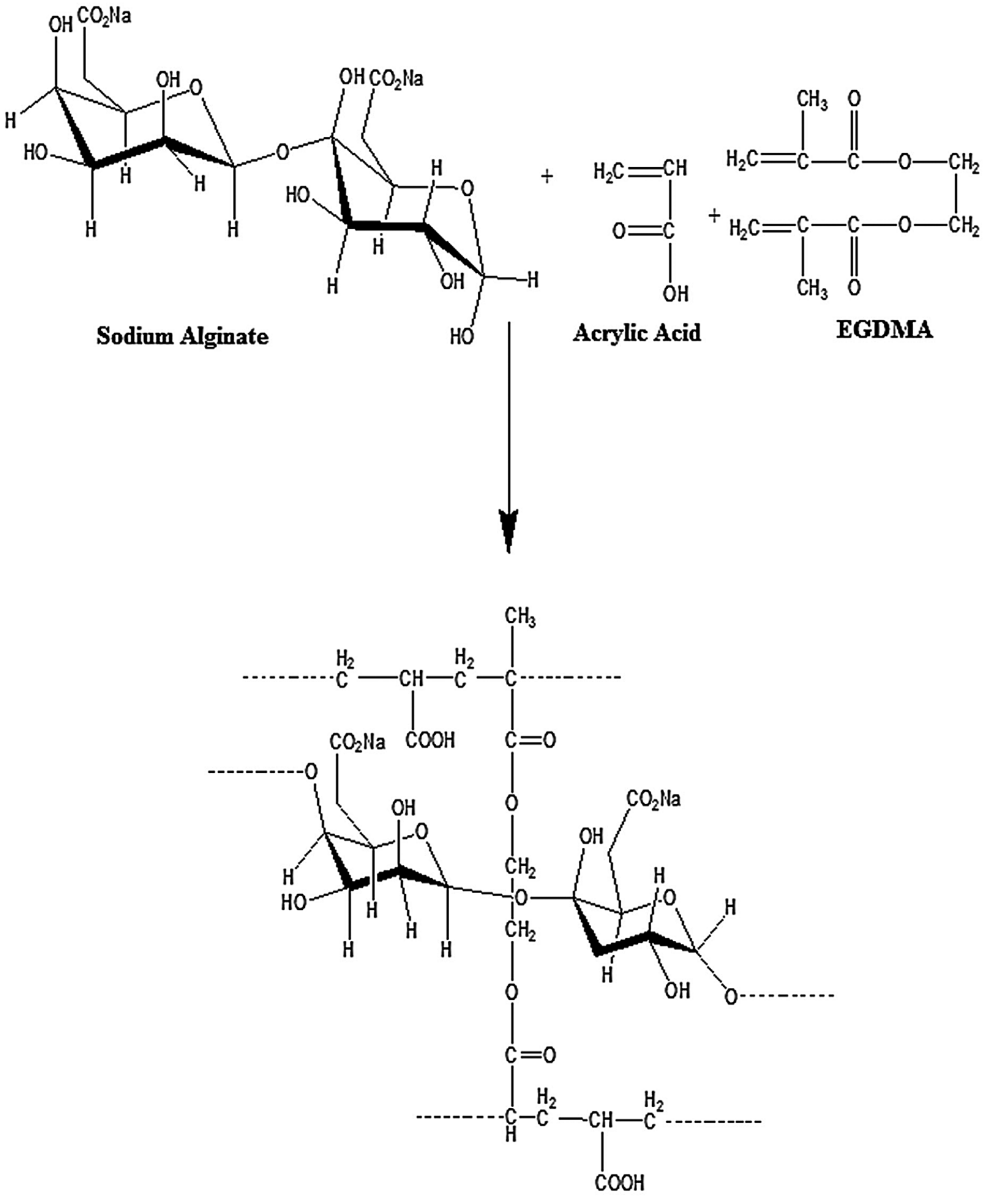
Presumptive structure of cross-linked NaAlg/AA hydrogels.

In the current work, a series of NaAlg/AA based porous hydrogels with various feed composition ratio were synthesized by free radical polymerization technique.[24] Table 1 indicates the formulation sheet of NaAlg/AA hydrogels. A weighed amount of sodium alginate was poured in pre calculated quantity of double distilled water under constant stirring for 45 min to get a clear solution. Ammonium per sulphate (APS) was taken as 1wt% of acrylic acid and dissolved in double distilled water under constant stirring to get a clear solution. Pre-determined amount of acrylic acid was weighed and ammonium per sulphate solution was added in acrylic acid and subject to stirring at 100 RPM. Calculated quantity of ethylene glycol methacrylate (EGDMA) was added drop wise into the acrylic acid solution with constant stirring. Now the prepared acrylic acid solution was added drop wise into the sodium alginate solution which was cooled after preparing a clear solution. During the addition of acrylic acid solution into the sodium alginate solution under constant stirring until a clear solution is obtained. The prepared homogenous mixture is then poured into glass tubes (Pyrex) having 16 mm internal diameter and 150 mm length. Nitrogen bubbling was done for 10 min to remove the air from the prepared mixture and immediately after bubbling the test tubes are capped to stop the incorporation of air. The capped test tubes are placed in a preheated water bath at 45 °C. A temperature scheme was given as 45 °C for one hour, 50 °C for two hours, 55 °C for three hours, 60 °C for four hours and 65 °C for twelve hours for the reaction to be completed. The capped test tubes are then cooled at room temperature and cylindrical hydrogels had been removed from the test tubes and cut into discs of 5 mm diameter. The discs were washed with the double distilled water on daily basis until the pH of the distill water became same as before washing. The hydrogel discs were washed in order to remove the unreacted crosslinker, monomer, polymer and initiator. After the washing was completed. The discs were allowed to dry at room temperature and then at 45 °C in oven. Figure 2 indicates the presumptive structure of cross-linked NaAlg/AA hydrogels.

**Table 1.** Formulation sheet of NaAlg/AA hydrogels.

Samples codes	NaAlg/100 g of solution	Acrylic acid/100 g of solution	AA/NaAlg (wt% ratio)	EGDMA/100 g of solution	EGDMA wt% of AA
S1	1.7	30	94.60/5.40	0.12	0.4
S2	2.1	30	93.45/6.55	0.12	0.4
S3	2.5	30	92.30/7.70	0.12	0.4
A1	2.1	26	91.95/8.05	0.12	0.4
A2	2.1	32	93.84/6.16	0.12	0.4
A3	2.1	38	94.47/5.53	0.12	0.4
E1	2.1	30	93.45/6.55	0.09	0.3
E2	2.1	30	93.45/6.55	0.15	0.5
E3	2.1	30	93.45/6.55	0.18	0.6

### Swelling characterization of synthesized hydrogels

#### Dynamic and equilibrium swelling studies

To perform the swelling studies USP phosphate buffer medium of varying pH of 1.2, 5.5, 6.5 and 7.5 were used to imitate the pH of gastric as well as of small intestine by keeping the same ionic strength. The oven dried discs which were obtained after washing and drying procedure were weighted and immersed in the different pH mediums (1.2, 5.5, 6.5 and 7.5) at room temperature. At predetermined intervals, swollen gels were removed and blotted with blotting paper then weighed and put in similar bath solution. The swelling was reported at time (*t*).The Dynamic swelling proportion of each hydrogel sample had been estimated from the following equation.

(1) $$q=\frac{W_{h}}{W_{d}}$$

*W*_*h*_ represents the weight of swollen gel at time *t*, and *W*_*d*_ represents the initial weight of dry disc of the hydrogel. The swelling procedure was continued in order to estimate the equilibrium proportion of the hydrogels and final calculation is obtained when the weight of the disc become constant. Equilibrium swelling ratio was calculated by using the following equation.

(2) $$S_{\text{(Eq)}}=\frac{W_{h}}{W_{d}}$$

Weight of the swelled gel at equilibrium is represented by *W*_*h*_ and initial weight of dry hydrogel disc by *W*_*d*_.[25]

#### Diffusion coefficient

The diffusion coefficient can be defined as the amount of substance diffusing across the unit area through a concentration gradient in the unit time. The following equation is used to estimate the water diffusion coefficient of hydrogels.[26]

(3) $$D=\pi {\left(\frac{h\cdot \theta }{4\cdot Q_{\text{eq}}}\right)}^{2}$$

where *D* = Diffusion coefficient of the hydrogels, *Θ* = slop of the linear part of the swelling curves, *h* = Initial sample thickness of the dried hydrogel disc, *Q*_eq _= Swelling of gel at equilibrium.

### Characterization of network structure of NaAlg/acrylic acid hydrogel

#### Solvent interaction parameters (χ)

The compatibility of polymer with the molecules of surrounding solution is evaluated by measuring the solvent interaction parameter. Polymer volume fraction is the amount of fluid imbibed and retained by the hydrogel in swollen state. Flory–Huggins equation is used to estimate the solvent interaction parameters (*χ*). Formula used to calculate *χ* values is given below

(4) $$\chi =\frac{\ln\left(1-v_{2,s}\right)+v_{2,s}}{v_{2,s}^{2}}$$

*v*_2,*s*_ (ml/mol) is the volume proportion of the swollen hydrogel *q*_eq_ and *χ* is the (Flory–Huggins polymer solvent interaction parameters).[27]

#### Molecular weight between crosslinks (M_c_)

NaAlg/AA hydrogels are also evaluated for molecular weight between the crosslinks by employing Flory-Rehner theory. According Flory there is gradual increase of *M*_*c*_ with the subsequent increase in the swelling ratio of hydrogels.[27,28] *M*_*c*_ is calculated by the following formula.

(5) Mc=-dpvsv2,s1/3-v2,s2ln1-v2,s+v2,s+xv2,s2

Volume fraction of the polymer *v*_2,*s*_ was estimated by the following equation;

(6) $$v_{2,s}={\left[1+\frac{d_{p}}{d_{s}}\left(\frac{M_{a}}{M_{b}}-1\right)\right]}^{-1}$$

where *d*_*p* _= Density (gm/ml) of the hydrogel, *d*_*s*_ = Density of solvent, *M*_*a* _= Maas of the hydrogel in swelled form, *M*_*b* _= Mass of the dry hydrogels, *v*_2,s _= (ml/mol) is volume fraction of the swelled hydrogel, *Χ* = Flory–Huggins polymer solvent interaction parameters.

#### Crosslinked density (q)

Crosslinking density is employed for description of cross-linked hydrogels.[29] The equation applied for crosslinked density is as follow

(7) $$q=\frac{M_{C}}{M_{r}}$$

where is molar mass (*M*_*r*)_ of the repeating unit and is calculated as

(8) $$M_{r}=\frac{m_{\text{SA}}M_{\text{SA}}+m_{\text{AA}}M_{\text{AA}}+m_{\text{EGDMA}}M_{\text{EGDMA}}}{m_{\text{SA}}+m_{\text{AA}}+m_{\text{EGDMA}}}$$

where *m*_SA_, *m*_AA_ and *m*_EGDMA_ are the feed masses of the polymer sodium alginate, acrylic acid and EGDMA respectively. While *M*_SA_, *M*_AA_ and *M*_EGDMA_ are the molar masses of sodium alginate, acrylic acid and EGDMA respectively.

#### Sol–gel analysis

Five-millimeter hydrogels discs were first dried at room temperature and then at 45 °C in oven until their weight became constant. The discs used for the purpose of sol–gel analysis were not subject to washing. Soxhelt extraction technique was used for the purpose of sol–gel analysis. Deionized water was used as solvent at boiling temperature for 4 h. During this process un reacted reactants (monomer, polymer and cross linker) were removed from the gel network. The gels were collected from the apparatus first dried at room temperature and at 45 °C in the oven until their weight became constant.[30] The gel fraction of the samples was calculated by employing following formula.

(9) $$\text{Sol}\;\text{fraction}\;\left(\%\right)=\left[\frac{W_{o}-W_{\text{ext}}}{W_{o}}\right]\times 100$$

(10) $$\text{Gel}\;\text{fraction}\;\left(\%\right)=100-\text{Sol}\;\text{fraction}$$

where *W*_0 _= weight of the dry hydrogel before extraction process, *W*_ext_ = weight of the hydrogel after extraction process.

#### Porosity measurement

Solvent replacement technique is employed to estimate porosity of the gels. Weighted dried hydrogels discs were first weight and then submerge in the absolute ethanol overnight after the time of overnight the gel discs were pulled from the absolute ethanol and then blotted with filter paper and their weight is noted. The porosity (%) was estimated by the employing this equation.[27]

(11) $$\text{Porosity}\;=\;\frac{\left(M_{h}-M_{d}\right)}{\rho V}\times 100$$

where *M*_*d* _= weight of the dried gel disc, *M*_*h* _= weight of hydrogel after submerging in absolute ethanol, *P* = Density of absolute ethanol, *V* = volume of hydrogel disc.

### Diclofenac potassium loading of crosslinked NaAlg/AA hydrogels

Samples were selected for the loading of drug as well as for release studies. Total six samples were selected out of which three samples have varying acrylic acid content (26, 32 and 38%) and remaining three have varying EGDMA concentration (0.3, 0.5 and 0.6%). Drug solution of diclofenac potassium was prepared by dissolving water soluble drug in doubled distill water. Weighted dry hydrogel disks were then placed in this drug solution up to their equilibrium swelling of drug loaded hydrogels. Hydrogels were dried primarily at room temperature and after then at 45 °C in the oven up to constant weight.[27]

### Determination of diclofenac potassium loading

Basically three methods were employed to estimate the percent drug loading in the hydrogels.

(1) By weight method.
(2) By extraction method
(3) By swelling method.

The first method is called dry weight method in which the amount of drug loaded is calculated by following equation.

(12) $$\text{Amount}\;\text{of}\;\text{drug}=W_{D}-W_{d}$$

where *W*_*d* _= weight of the dried drug unloaded gel disc, *W*_*D* _= weight of dried drug loaded gel disc.

Percent drug loading was calculated by the following formula.

(13) $$\text{Drug}\;\text{loading}\;\%\;=\;\frac{W_{D}-W_{d}}{W_{d}}\times 100$$

In second method the loaded drug is calculated by continuously extracting the drug from the hydrogels by replacing the solvent on daily basis until all the drug in the hydrogel is extracted and calculated. In this method every time 25 ml fresh doubled distilled water solution was employed for extraction of drug from the hydrogels until there was no drug in the solution. Spectrophotometric technique was used to estimate the concentration of the drug in the solution on daily basis. In last the amount of drug present in each portion of the extracts was summed up and in this way total amount of the loaded drug is estimated.

In the third method the amount of drug loaded in the hydrogel is calculated by measuring the weight of the gel in its equilibrium swelling state. At first the initial dry weight of the hydrogel is noted down and then the weight after gaining the equilibrium swelling state was noted down. The difference between the two weight is calculated to estimate the weight of the drug solution entrapped in the hydrogel. Total Volume of solution of drug absorbed by gel disc can be estimated via density as well as from weight of drug solution. The amount of drug absorbed by gel disc was calculated.[30]

### Diclofenac potassium release studies in vitro

Drug release was calculated by measuring the dissolution in dissolution apparatus (Pharma test; PT-Dt 7) and with UV–Vis spectrophotometer (Perkin Elmer Lambda 25).The weighted hydrogel disc was placed in 900 ml dissolution medium at 37 °C and dissolution medium was set at a rate of 100 rpm for maintaining a uniform drug concentration in the medium. 0.05 M USP phosphate buffer solutions of pH 1.2, 5.5 and 7.5 were used for dissolution medium. Diclofenac potassium released studies were conducted at *λ*_max_ 282 nm up to 24 h after regular intervals. Each time 5 ml solution was drawn for UV analysis and drawn amount is compensated by fresh USP phosphate buffer solution.[31]

### Analysis of drug release pattern

Diclofenac potassium loaded hydrogel are also analyzed for the purpose of evaluating drug release behavior. 0th-order, 1st-order, Korsmeyer–peppas as well as Higuchi models are used. The four models are applied to fully understand the release kinetic of the drug by hydrogels. When more than one phenomena is involved in drug release kinetics then usually we used these models. Peppas equation is mostly used to evaluate the release profile by employing peppas semi-empirical equation of peppas. Following models are used for release calculations.

(14) $$0\text{th}-\text{order kinetics}:\;F_{t}=\;K_{0}t$$

where *F* denoted for the proportion of drug release in time *t* and *K*_0_ is the zero-order release constant.

(15) $$1\text{st-order kinetics:}\;\ln\;\left(1-F\right)=-K_{1}t$$

where *F* denoted for the proportion of drug release in time *t* and *K*_1_ is the 1st-order release constant.

$$\text{Higuchi}\;\text{model}:\;F=K_{2}t1\;/\;2$$

where *F* denoted for the proportion of drug release in time *t* and *K*_2_ is the Higuchi constant.

(17) $$\text{Korsmeyer}–\text{Peppas}\;\text{model:}\;M_{t}/M\propto =K_{3}t^{n}$$

where *M*_*t* _= mass of water absorbed at any time t or penetrant time *t*, *M*∝ = amount of water at equilibrium or mass uptake at equilibrium, *K*_3_ = kinetic constant, *n* = the exponent describing the swelling mechanism.

If *n* = 0.45 or greater than the release mechanism is called as non-fickian.[32–34]

### FT-IR spectroscopic analysis

The dried hydrogel discs were powdered with the help of pestle and mortar. The crushed material was mixed with potassium bromide in 1:100 proportions was mixed with the powdered hydrogel discs, placed in oven for drying at 40 °C. The mixture was compressed to a 12 mm. A semitransparent disc size of 12 mm was prepared from powdered mixture with the help of a pressure of 65 kN (Pressure gauge, Perkin elmer) for 3 min. the samples were run on whole range of FT-IR spectrum from 4500–400 cm^−1^ were recorded using FTIR spectrometer (FT-IR 8400 S, Shimadzu). The FT-IR spectrum of the pure polymer, monomer and drug was also analyzed by same procedure.[27]

### Differential scanning calorimetry

DSC was performed to characterize hydrogel for thermal stability. DSC was done in the DSC unit (Netzsch DSC-200 PC Phox, Germany).The samples were heated in a close aluminum pan at the rate of 40 °C/min under nitrogen flow (50 mL min^−1^).[23]

### XRD pattern analysis

Bruker D8 Discover (Germany) instrument was used for the analysis of XRD patterns were recorded for drug loaded and unloaded hydrogels. The instrument was operated at at 25 °C temperature, using the nickel filtered CuKα radiation (*λ* = 1.54050 Å) and operating at voltage (40 kV), and current (35 mA), step scan 0.1° of 2*θ* and 1 s of counting time. Eva software was used for the data processing (Evaluation Package Bruker, Germany). The range of diffraction angle was 10° **−** 70° 2*θ*.[27]

### Scanning electron microscopy

Surface and cross-sectional morphology of unloaded IPN samples and drug-loaded samples were investigated using SEM (440, Leica Cambridge Ltd., Cambridge, UK). Hydrogel samples were mounted on an aluminium mount and sputtered with gold palladium. The specimen mounts were then coated with 60% gold and 40% palladium for 30 s with 45 mA current in a sputter coater (Desk II, Denton Vacuum, Moorestown, NJ, USA). The coated specimens were then observed on SEM using an accelerating voltage of 20 kV at a tilt angle of 30° to observe the microstructure of the sample.[11]

### Statistical analysis

For the statistical analysis of data, Student’s *t*-test has been applied to compare the results and to determine the statistical significant/non-significant interpretation at 95% confidence interval, *p*-value less than 0.05 was considered as significant difference in results.

## Results and discussion

### Effect of pH on swelling and drug release from NaAlg/AA hydrogels

Swelling changes at various pH values have been observed in hydrogels containing carboxylic groups because the swelling behavior of gel systems is an important factor to assess pH sensitivity and also to understand the diffusion mechanism. The ionization of hydrogel depends mainly upon the pH of the buffer solution as well as on the pKa of the hydrogel carboxylic group. The best results of swelling of the hydrogel are obtained when the pH of the buffer medium is above the pKa of the carboxylic acid present in the gel. When the pH of the buffer medium is above the pKa of the gels then the buffer medium accept the protons and ionize the gels. This experiment was designed to evaluate the synthesized hydrogels swelling capability in buffer solutions (0.05 M USP phosphate buffer solutions of pH 1.2, 5.5, 6.5 and 7.5). The dynamic and equilibrium swelling ratios of NaAlg/AA hydrogels is given in Table 2. As the charge balance inside the gelling network changes in accordance with the pH of the medium which also influence the degree of interaction between NaAlg and acrylic acid thus swelling occurs because of the dissociation. The hydrogels in solution of pH 7.5 show maximum swelling. At acidic pH the carboxylate groups of the gels are deprotonated and the similar charge repulsive forces are abolished and decreased swelling pattern is observed. At basic pH the ionization of the carboxylic group starts and increase swelling pattern is observed due to the electrostatic repulsion between –coo^−^ groups.[35]

**Table 2.** Dynamic (*q*) and equilibrium swelling (Eq) ratios of NaAlg/AA hydrogels.

Sample no	pH 1.2		pH 5.5		pH 6.5		pH 7.5	
	*q*	Eq	*q*	Eq	*Q*	Eq	*q*	Eq
S1	2.32	5.03	10.53	55.17	11.88	78.84	13.50	84.19
S2	2.45	5.25	11.06	59.36	12.24	79.49	13.42	86.43
S3	2.52	5.41	11.40	64.40	12.59	86.21	13.86	90.43
A1	2.54	5.81	12.14	75.59	13.066	89.89	13.71	95.12
A2	2.29	4.76	10.06	49.23	10.90	69.27	12.02	81.12
A3	2.01	4.15	8.96	46.28	10.35	58.47	11.27	72.58
E1	2.33	4.93	8.89	60.18	9.75	67.96	10.67	78.50
E2	2.31	4.77	8.71	54.70	9.20	59.27	9.70	68.22
E3	2.03	4.66	8.47	50.48	8.96	52.35	9.40	66.91

Diclofenac potassium is freely soluble in water and was selected as a model drug. Buffer solutions of pH 1.2, 5.5 and 7.5 as used to investigate the drug release behavior of diclofenac potassium. Table 3 indicates the amount of model drug loaded and released (%) from selected samples. At higher pH (7.5) all the samples exhibit maximum drug release. It was the same behavior of the hydrogel which they had demonstrated in the swelling studies that with increase in the pH value of the buffer medium from acidic to basic the swelling increases. Wang Q et al., and his research fellows reported the similar release behavior of diclofenac in their experimental work from acrylic acid based hydrogels.[12]

**Table 3.** Amount of diclofenac potassium loaded and released (%) in different formulation of NaAlg/AA hydrogels.

Sample codes	Amount of diclofenac potassium Loaded (g/g of dry gel)			Amount of diclofenac potassium released (%) (pH of the solution)		
	By swelling	By weight	By extraction	1.2	5.5	7.5
A1	0.362	0.365	0.358	15.61	56.57	79.48
A2	0.319	0.311	0.305	14.15	54.66	75.21
A3	0.289	0.281	0.273	13.40	51.20	73.09
E1	0.294	0.286	0.283	16.35	57.56	82.79
E2	0.227	0.229	0.220	11.88	52.92	75.36
E3	0.201	0.197	0.206	10.26	51.11	73.42

### Effect of acrylic acid concentration on swelling and on drug release of NaAlg/AA hydrogels

**Figure 3.**
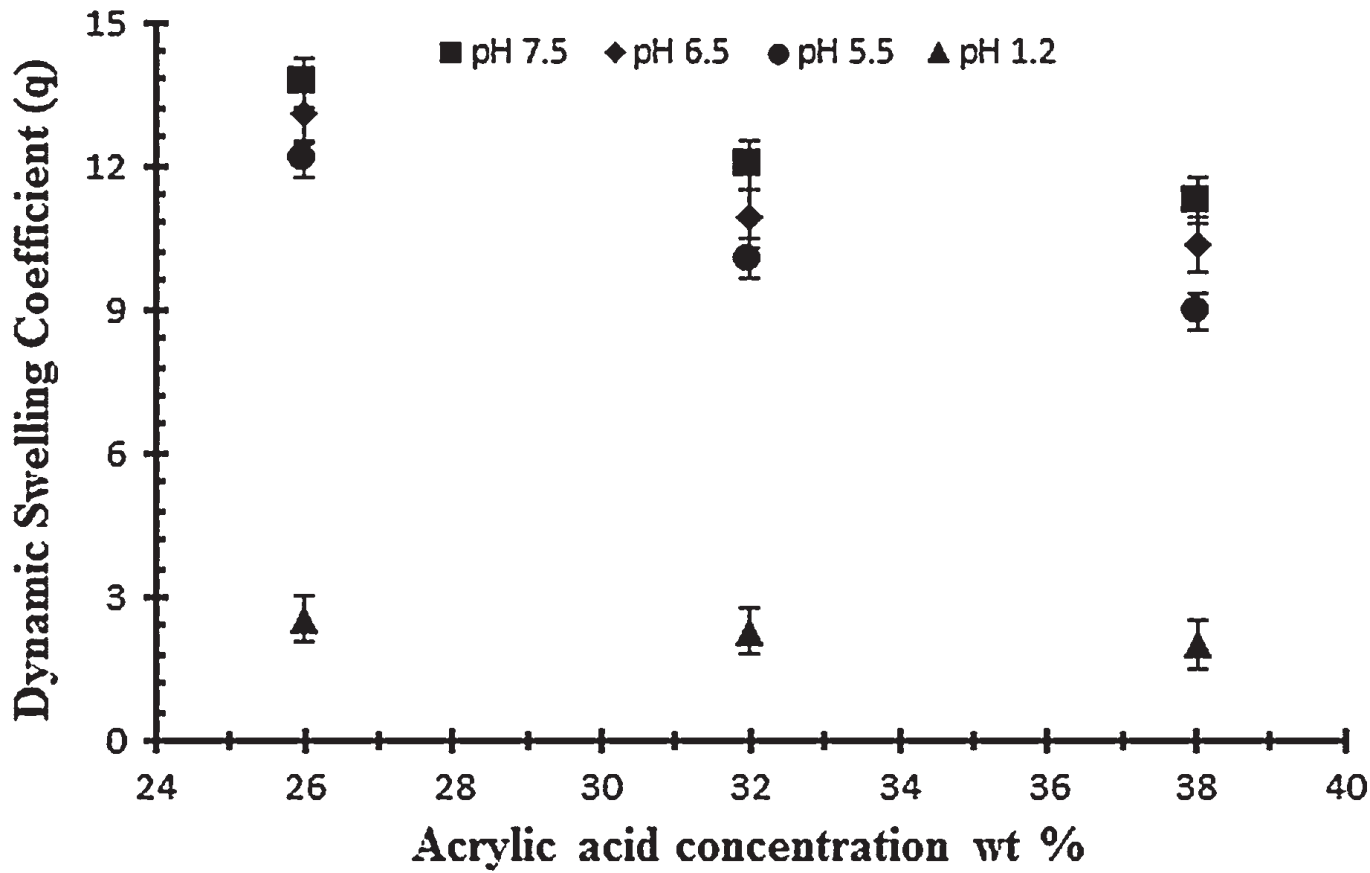
Dynamic swelling ratio (*q*) of NaAlg/AA hydrogels with different concentrations of AA (26, 32 and 38 g) using EGDMA as crosslinking agent (0.4 wt%) in solution of different pH in 0.05 M USP phosphate buffer.

To study the effect of monomer on swelling and drug release, AA was employed at different concentration of 26, 32 and 38 g per 100 g of solution keeping the polymer and EGDMA content constant (0.4 wt% of acrylic acid). Figure 3 shows the effect of acrylic acid on the dynamic swelling pattern of hydrogel samples. Normally by increasing the concentration of AA in the formulation there is increased swelling but in the present work it is contrary. In the experiment it is observed that by increasing the AA content the swelling decreases. In Table 2, the samples with codes A1, A2 and A3 show the dynamic and equilibrium swelling ratio. This contrary effect could be attributed to the fact that AA is a small molecule and increasing the content causes smaller nodes with increase in cross-linking and decrease of cross-link density. So these factors contribute to low swelling of the hydrogel by increasing concentrations of AA. This is further confirmed by studies on structural parameters of hydrogels. Secondly, increased AA content may influence the preference of homopolymerization over copolymerization thus swelling ratio decreased at higher AA content.[36,37]

**Figure 4.**
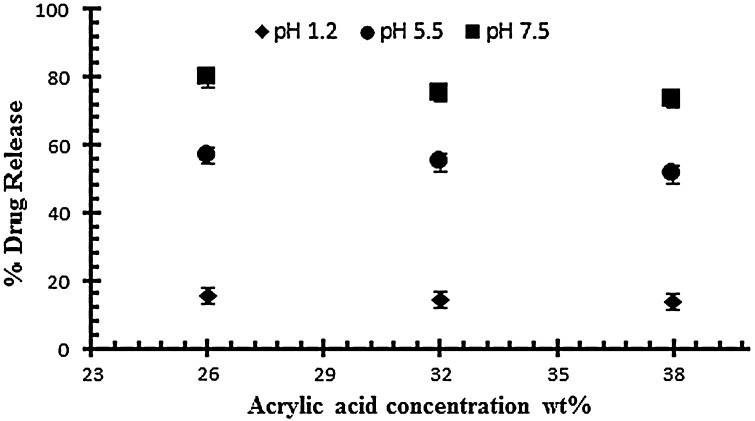
Release of diclofenac potassium from NaAlg/AA hydrogels using different concentrations of AA (26, 32 and 38 g) at various pH values in 0.05 M USP phosphate buffer.

The effect of acrylic acid concentration on drug release has also been investigated at different pH 1.2, 5.5 and on 7.5. The effect of acrylic acid ratio on % drug release of diclofenac potassium from drug loaded gels have been shown in Figure 4. The drug release from hydrogel at pH 7.5 is higher than as compared to pH 1.2 and 5.5. The swelling and drug release of NaAlg/AA hydrogel increases as the pH moves from acidic to basic. From the result it was observed that the percent drug release decreases by increasing the concentration of AA.

### Effect of NaAlg concentration on swelling of NaAlg/AA hydrogels

**Figure 5.**
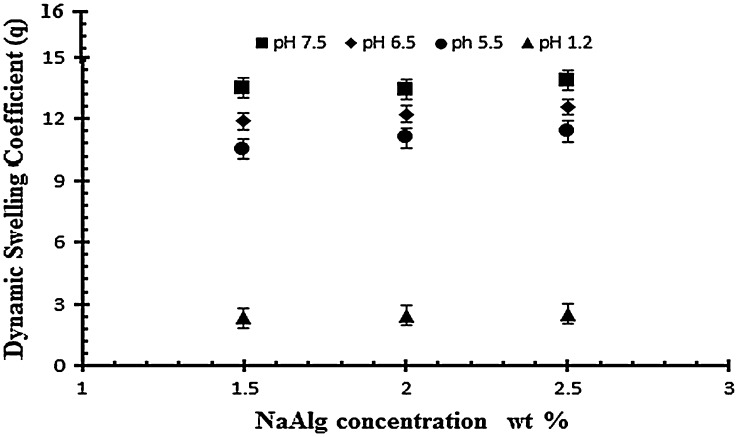
Dynamic swelling ratio (*q*) of NaAlg/AA hydrogels with different concentrations of NaAlg (1.5, 2 and 2.5 g) using EGDMA as crosslinking agent (0.4 wt%) in solution of different pH in 0.05 M USP phosphate buffer.

For the evaluation of polymer effect three formulations with different NaAlg concentrations of 1.50, 2 and 2.5 g per 100 g of the solution, keeping the concentration of AA and EGDMA concentration constant (0.4 wt% of acrylic acid). Na alginate is a pH sensitive polymer because of integrated carboxyl groups in its structure. In Table 2 samples (S1, S2 and S3) show the effect of NaAlg on the dynamic and equilibrium swelling ratio of NaAlg/AA hydrogel. The results shows that there is not a significant swelling at low pH because the NaAlg has carboxyl group and due to their protonation in the acidic pH the anion repulsive forces were negligible and therefore swelling was not much significant. While at higher pH (pH > 4) the samples shows a significant swelling due to the ionization of the carboxyl group which strengths the similar charge repulsive forces. It is observed that by increasing the amount of NaAlg the swelling of gel increases. Mohammad et al., and Hua S et al., and their research fellows reported the similar swelling behavior of sodium alginate in their experimental work.[38] The swelling behavior of the sodium alginate in different ratio and in varying pH buffer medium is shown in the Figure 5.

### Effect of degree of crosslinking on swelling and on drug release of NaAlg/acrylic acid hydrogels

**Figure 6.**
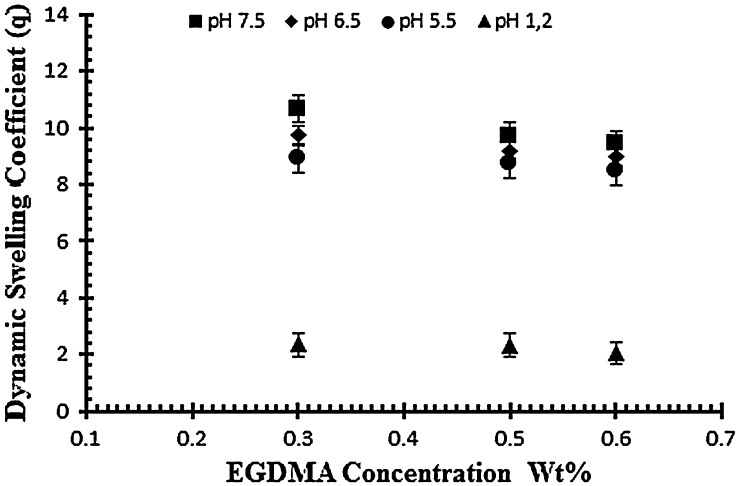
Dynamic swelling ratio (*q*) of NaAlg/AA hydrogels with different concentrations of EGDMA (0.3, 0.5 and 0.6 wt%) as crosslinking agent in solution of different pH in 0.05 M USP phosphate buffer.

To study the effect of cross linker (EGDMA) three hydrogel samples (E1–E2) are subjected to study the swelling pattern with different concentration of cross linker (0.3, 0.5 and 0.6 wt% of acrylic acid) at different pH values ranging from 1.2 to 7.5. Samples with codes E1–E3 represents the effect of crosslinking agent on dynamic and equilibrium swelling ratio as shown in Table 2. The results reveal that the swelling decreases as the concentration of cross linker increases. By increasing EGDMA the mesh size of the hydrogel network decreases and there is increase in the stability of the hydrogel network. The stability of the network increases because the cross linker tightens the structure more as its concentration increases and results in the low swelling of the hydrogels. The mobility of the polymer chain is also affected by cross linker because it hinders the mobility of the polymer chain which ultimately results in low swelling. Figure 6 shows the effect of cross linker on the swelling of the hydrogel by keeping the monomer and polymer concentration constant.[31]

**Figure 7.**
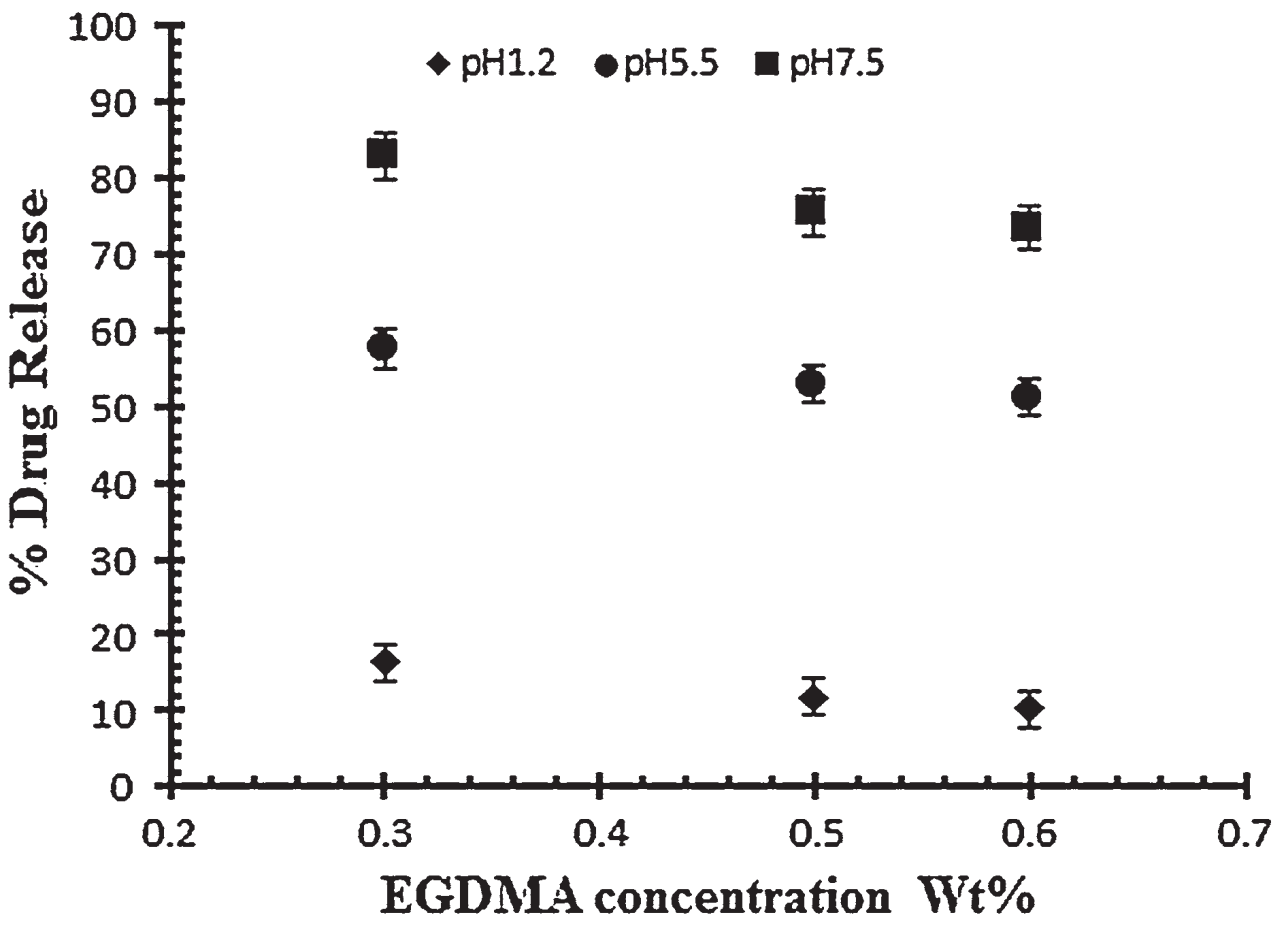
Release of diclofenac potassium from NaAlg/AA hydrogels using different concentrations of EGDMA as crosslinking agent (0.3, 0.5 and 0.6 various pH values in 0.05 M USP phosphate buffer.

The drug release study was also investigated as a function of increasing EGDMA concentration in buffer solution of different pH values i.e. pH 1.2, 5.5 and 7.5. Table 3 indicates the % drug release of samples with increasing EGDMA concentration. It was observed that with the increase in EGDMA concentration drug release decrease as shown in Figure 7. The decrease in drug release is mainly attributed due to tightens structure of gel network.[11]

### Diffusion coefficient of polymers (D)

The measure of the solute diffusion into the hydrogel is measure by diffusion coefficient. Flick’s first law of diffusion is usually used to measure the permeation of the solute into the xerogel. Diffusion coefficient increased with increasing the feed concentration of the acrylic acid and crosslinker EGDMA because the swelling of the polymer decrease on increasing the amount of both. Diffusion coefficient decreased by increasing the concentration of the sodium alginate because the swelling increases gradually on increasing the sodium alginate concentration.[11] Table 4 indicates the value of diffusion coefficient of porous hydrogels

**Table 4.** Flory–Huggins network parameter of NaAlg/AA hydrogels.

Samples codes	*v*_2,*s*_	*χ*	*M*_*c*_	*M*_*r*_	*q*	*D* (cm²/sec)
S1	0.247735	−0.60176	72.12	79.30	0.909	0.381591
S2	0.22126	−0.58865	108.78	81.43	1.33	0.348889
S3	0.238343	−0.59703	87.60	83.49	1.049	0.327951
A1	0.199746	−0.57847	121.99	80.36	1.15	0.208936
A2	0.213217	−0.5848	100.33	78.48	1.27	0.31077
A3	0.22872	−0.59228	101.54	77.83	1.43	0.558354
E1	0.210174	−0.58336	138.27	79.20	1.39	0.395282
E2	0.219274	−0.5877	113.81	79.42	1.43	0.309341
E3	0.222695	−0.58935	110.99	79.53	1.74	0.325175

### Molecular weight between crosslinks (M_c_) and solvent interaction parameters (χ)

A gradual decrease in the values of molecular weight between the crosslink’s was observed on increasing the concentration of the acrylic acid while an increase trend is observed with the increase of the sodium alginate in hydrogel network. Higher swelling was noted due to polymer sodium alginate carboxyl groups in polymer chain. Crosslink density is also related to the values of the acrylic acid and sodium alginate and molecular weight between the crosslinks. To evaluate the interaction between the polymer and solvent, solvent interaction parameters were also evaluated. It had been reported that higher the values of the (*χ*) weaker will be the interaction between polymer and solvent.[39] Table 4 indicates the values of Molecular weight between crosslinks (*M*_*c*_) and solvent interaction parameters (*χ*).

### Sol–gel analysis

**Figure 8.**
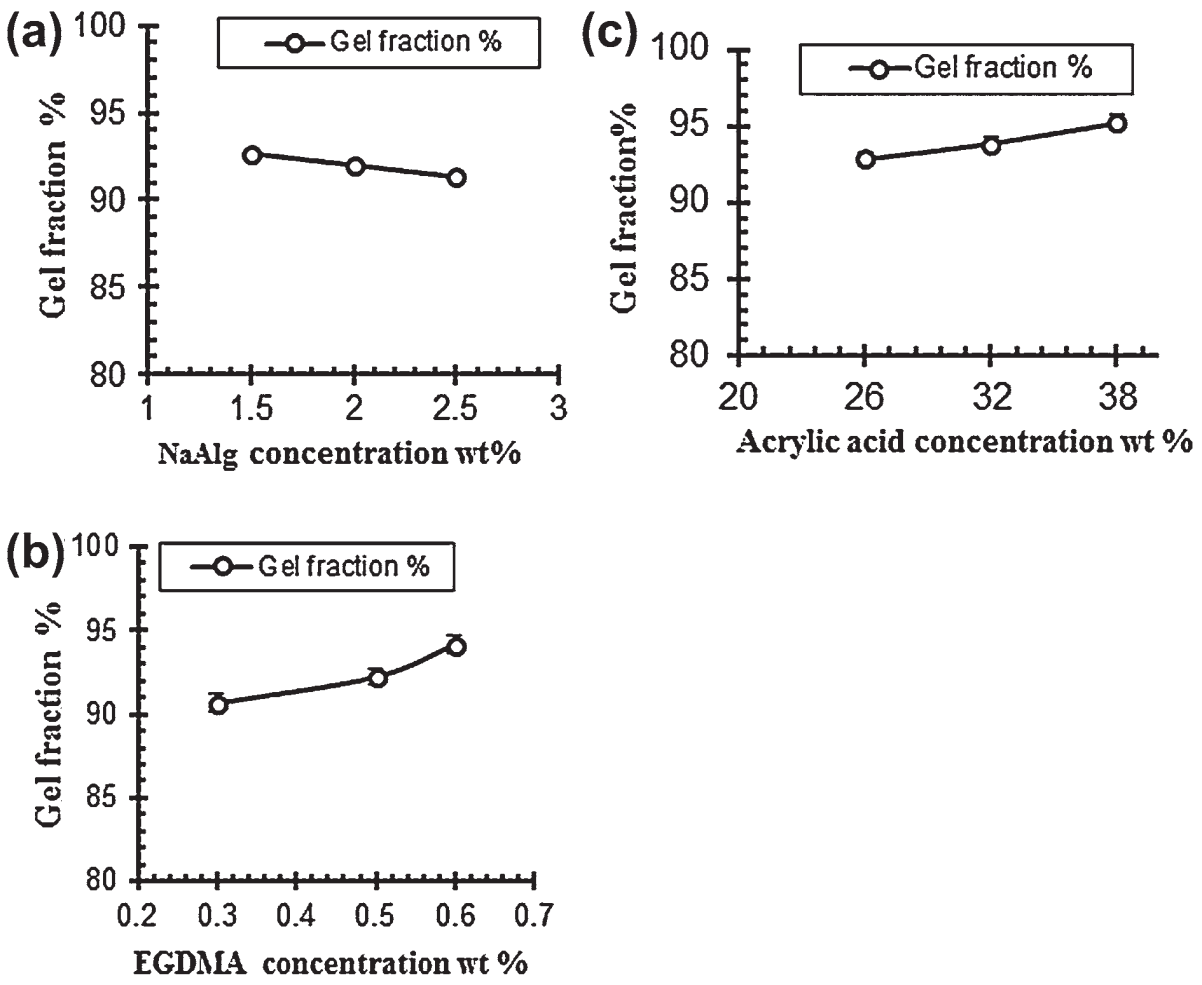
Effect of variable concentration on gel fraction (a) NaAlg concentration (b) Acrylic acid concentration and (c) EGDMA concentration.

To determine the uncrosslinked fraction of the polymer in hydrogels sole-gel analysis was performed. Table 5 indicates the gel fraction of NaAlg/AA hydrogels. The data obtained from the analysis revealed that the gel fraction increased with the increased of acrylic acid (AA) and cross linker ethylene glycol methylacrylate (EGDMA). While the sol fraction of the samples decreased with the increased of the AA and EGDMA concentration. The gel fraction decreased with the increasing concentration of the sodium alginate while the sol fraction increased as shown in Table 5.[31] Figure 8 shows the effect of polymer, monomer and cross linker concentration on gel fraction of the hydrogels.

**Table 5.** Effect of reaction variables on gel fraction and porosity.

Sample codes	Degree of crosslinking EGDMA (wt%)	Gel fraction (%)	Porosity (%)	*p***-**Value
S1	0.4	92.66	24.31	0.005
S2	0.4	91.98	26.08	0.05
S3	0.4	91.31	29.86	0.05
A1	0.4	92.83	22.14	0.005
A2	0.4	93.85	25.07	0.005
A3	0.4	95.20	27.35	0.05
E1	0.3	90.36	27.11	0.005
E2	0.5	91.17	23.97	0.005
E3	0.6	93.33	22.82	0.005

### Porosity measurement

**Figure 9.**
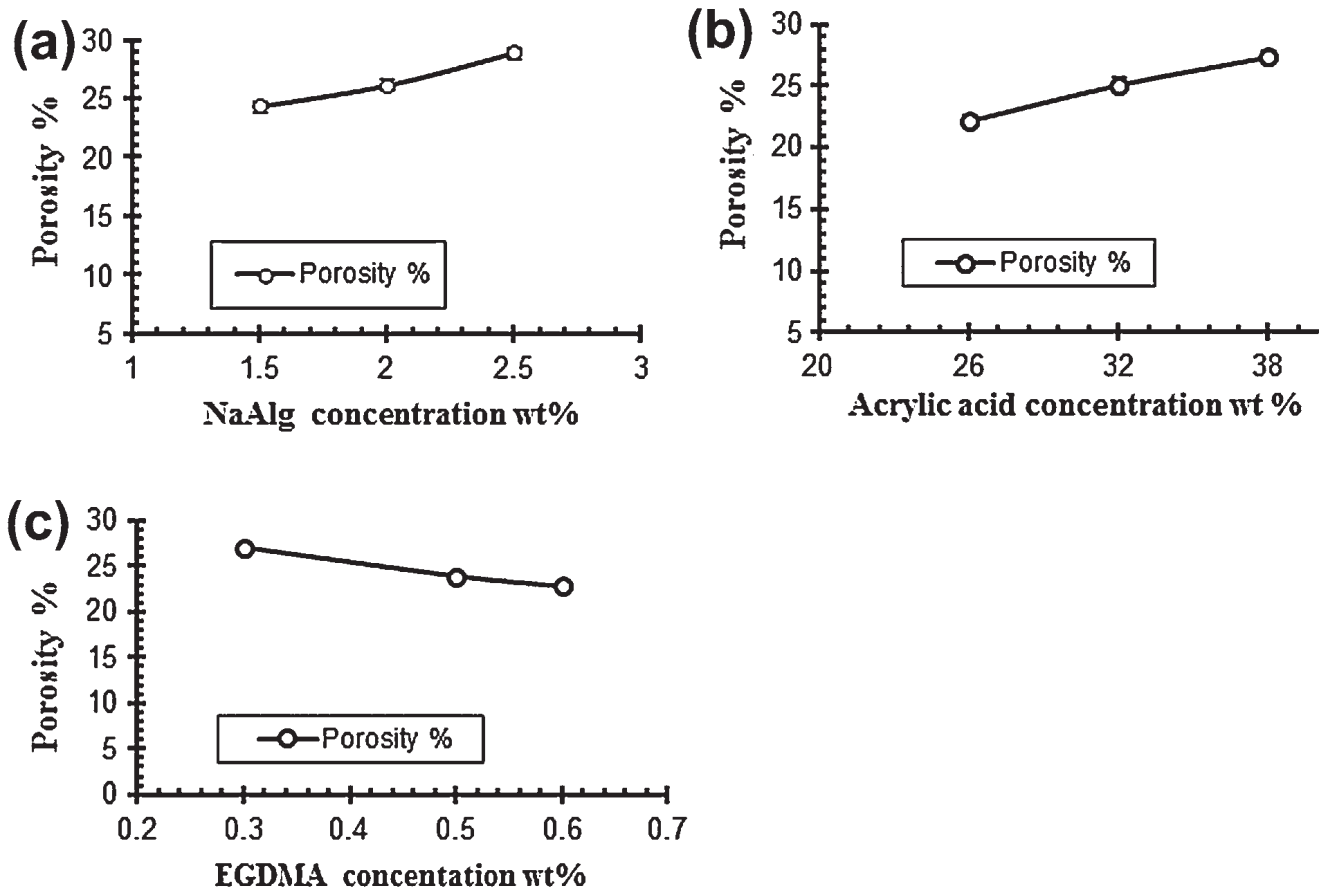
Effect of variable concentrations on porosity % (a) NaAlg concentration (b) Acrylic acid concentration and (c) EGDMA concentration.

Porosity measurement is done to check the porous nature of the hydrogels. The porosity values are given in the Table 5. It is evaluated from the results that the porosity of the hydrogels increased by increasing the concentrations of both acrylic acid and sodium alginate. It is due to increase in the viscosity of solution of the polymers because as the concentration of the polymers increases it prevents the escape of bubbles from the solution. These bubbles present in the structure of the hydrogel contribute towards the porosity of the hydrogels. On the other side by increasing the concentration of the cross linker EGDMA the porosity of the hydrogel decreases gradually because of increase in the physical entanglement between the polymers. Figure 9 show the effect of reaction variables on porosity for different samples of sodium alginate and acrylic acid.[40]

### Drug release mechanism

The penetration of water into the hydrogel network causes the release of drug from the drug loaded hydrogel. The hydrogel imbibe water and as results swells up and at that instance dissolution of drug occurs inside the hydrogel. After that the drug diffuse out through the aqueous channels to the surface of the drug delivery device. The swelling pattern of the hydrogels and drug release from the hydrogel has a direct relationship with each other. Regression coefficient (*r*) is mainly employed for the evaluation of the model that is most suitable for release kinetics of the delivery device. The most suitable model is selected on the basis of regression coefficient values (*r*) and defines the drug release behavior of the delivery device.

Zero order, first order, Higuchi and Pappas model were used to evaluate the value of regression coefficient (*r*) and release constant (*k*). Tables 6 and 7 shows values of ‘r’ from both kinetics. Drug loaded hydrogels of NaAlg were subject to dissolution studies to obtain the values of ‘r’ for zero order and first order. The experimental data reveal that the most of the samples follow zero order release kinetics model.

**Table 6.** Effect of different concentration of acrylic acid on drug release kinetics of NaAlg/AA hydrogels in solutions of different pH values using EGDMA as crosslinking agent (0.4% of AA).

Sample codes	AA contents	pH	Zero order kinetic		First order kinetics		Higuchi model	
			*K*_0_ (*h* − 1)	*r*	*K*_0_ (*h* − 1)	*r*	*K*_0_ (*h* − 1)	*r*
A1	26	1.2	1.283	0.998	0.014	0.997	0.054	0.987
		5.5	4.027	0.991	0.066	0.974	0.171	0.964
		7.5	5.648	0.997	0.115	0.956	0.240	0.970
A2	32	1.2	1.204	0.997	0.013	0.995	0.050	0.951
		5.5	3.710	0.983	0.053	0.957	0.157	0.951
		7.5	0.523	0.993	0.088	0.961	0.221	0.957
A3	38	1.2	1.148	0.992	0.012	0.986	0.044	0.969
		5.5	3.572	0.987	0.051	0.974	0.153	0.964
		7.5	5.075	0.991	0.093	0.947	0.214	0.954

**Table 7.** Effect of degree of crosslinking on drug release kinetics of NaAlg/AA hydrogels in solutions of different pH values.

Sample codes	EGDMA contents (%)	pH	Zero order kinetic		First order kinetics		Higuchi model	
			*K*_0_ (*h* − 1)	*r*	*K*_0_ (*h* − 1)	*r*	*K*_0_ (*h* − 1)	*r*
E1	0.3	1.2	1.340	0.997	0.014	0.995	0.245	0.968
		5.5	4.04	0.990	0.061	0.973	0.172	0.971
		7.5	5.075	0.991	0.093	0.947	0.214	0.954
E2	0.5	1.2	1.102	0.975	0.011	0.946	0.041	0.839
		5.5	3.780	0.991	0.054	0.975	0.160	0.959
		7.5	5.265	0.994	0.100	0.952	0.223	0.952
E3	0.6	1.2	0.983	0.970	0.009	0.966	0.038	0.883
		5.5	5.166	0.983	0.050	0.970	0.152	0.958
		7.5	5.166	0.993	0.095	0.958	0.219	0.964

By applying higuchi model on the drug release data reveals that the samples follow diffusion controlled drug release behavior. The linear values of the plot of drug release versus the square root of time reveals diffusion controlled system.

Tables 8 and 9 shows the effect of AA and EGDMA on release exponent (*n*). The slope and intercept of the plot ln *M*_*t*_/*M*_*α*_ versus ln *t* was used to calculate the value of ‘n’ for the release of diclofenac potassium at different pH (1.2, 5.5 and 7.5) and results shows that the value of ‘n’ is between 0.45 and 1.0. It reveals that the anomalous or non-fickian diffusion mechanism along with the swelling and relaxation of polymer are involved in drug release mechanism.

**Table 8.** Effect of different concentration of acrylic acid on drug release mechanism of NaAlg/AA hydrogels in solutions of different pH values using EGDMA as crosslinking agent (0.4% of acrylic acid).

Sample codes	AA content	pH	Release exponent (*n*)	*r*	Order of release
A1	26	1.2	0.808	0.998	Non-fickian
		5.5	0.650	0.992	Non-fickian
		7.5	0.589	0.986	Non-fickian
A2	32	1.2	0.957	0.993	Non-fickian
		5.5	0.629	0.992	Non-fickian
		7.5	0.581	0.980	Non-fickian
A3	38	1.2	0.898	0.992	Non-fickian
		5.5	0.651	0.991	Non-fickian
		7.5	0.582	0.980	Non-fickian

**Table 9.** Effect of degree of crosslinking on drug release mechanism of NaAlg/AA hydrogels in solutions of different pH values.

Sample codes	EGDMA content (%)	pH	Release exponent (*n*)	*r*	Order of release
E1	0.3	1.2	0.823	0.989	Non-fickian
		5.5	0.632	0.988	Non-fickian
		7.5	0.573	0.983	Non-fickian
E2	0.5	1.2	0.675	0.734	Non-fickian
		5.5	0.664	0.986	Non-fickian
		7.5	0.595	0.984	Non-fickian
E3	0.6	1.2	0.802	0.983	Non-fickian
		5.5	0.598	0.970	Non-fickian
		7.5	0.650	0.986	Non-fickian

### FTIR spectroscopy

**Figure 10.**
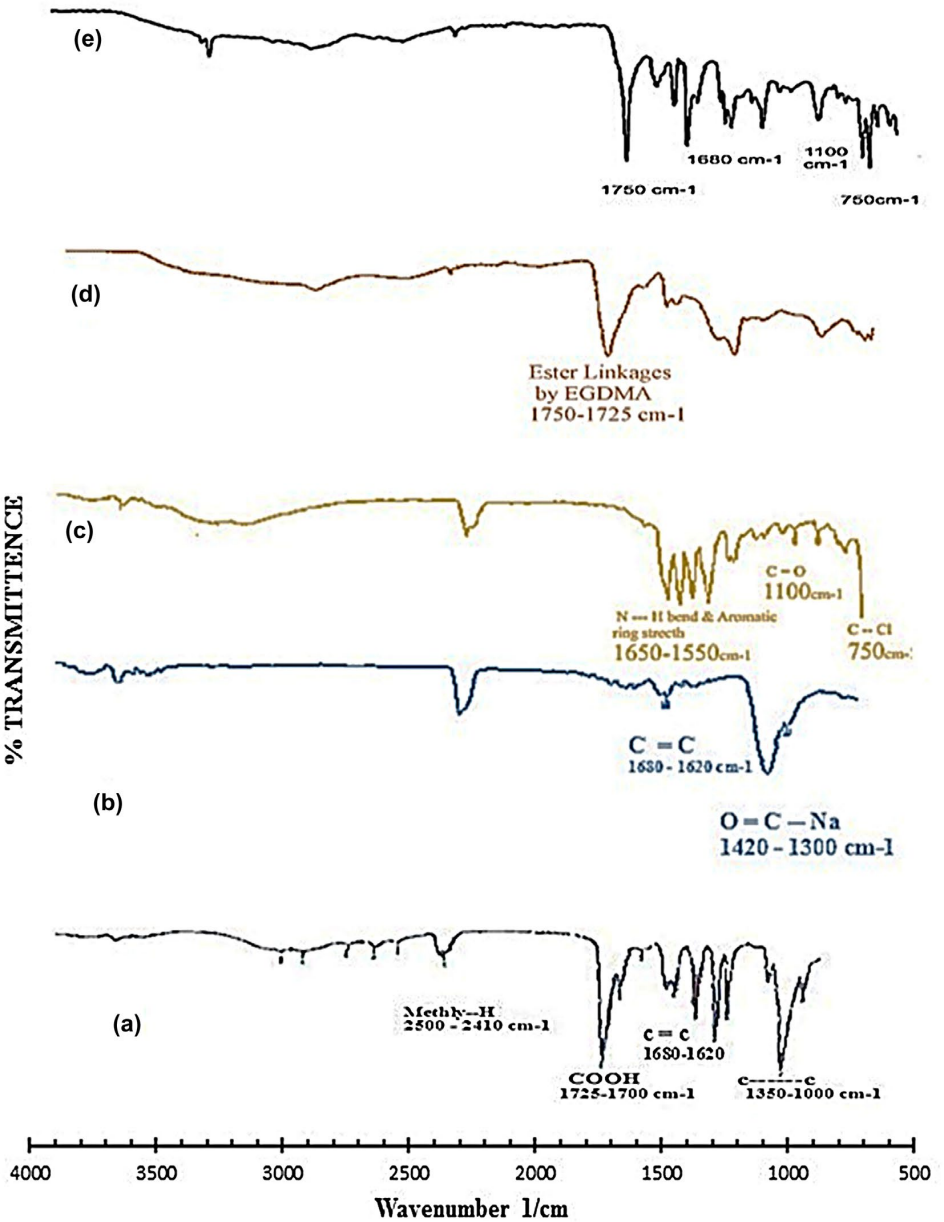
FTIR spectra of (a) acrylic acid (b) sodium alginate (c) diclofenac potassium (d) unloaded hydrogel and (e) loaded hydrogel.

In order to confirm NaAlg/AA interactions, samples were analyzed by FTIR. Figure 10 shows the FTIR spectra’s of pure AA, NaAlg, diclofenac potassium, NaAlg/AA unloaded hydrogel and drug loaded NaAlg/AA hydrogel. The FTIR of pure NaAlg showed peak at 3415 cm^−1^ due to the stretching of –OH groups. The peak at 2913 cm^−1^ indicated –C–H stretching vibration. The peak at 1751.92 cm^−1^ indicated >C=O stretching vibrations due to the presence of –COOCH_3_ group. The peaks at 1441 and 1342 cm^−1^ could be assigned to –CH_2_ scissoring and –OH bending vibration, respectively. The peak at 1150 cm^−1^ suggested the presence of –CH–OH group. The peak at 1023 cm^−1^ suggested –CH–O–CH– stretching.

The FTIR spectra of NaAlg/AA hydrogel indicated the main changes in the region of 1300–1800 cm^−1^ which is evidence of interaction between them also can be attributed to bonds overlapping. Incorporation of AA was confirmed by the formation of an extra peak at 1732 cm^−1^ for the carbonyl of acrylic acid units. The intensity of the esterified carboxyl group at 1751.92 cm^−1^ which is typical of NaAlg, decreased and shifted to a lower frequency i.e. 1742.63 cm^−1^ following interaction between NaAlg and acrylic acid**.**

### Differential scanning calorimetry

**Figure 11.**
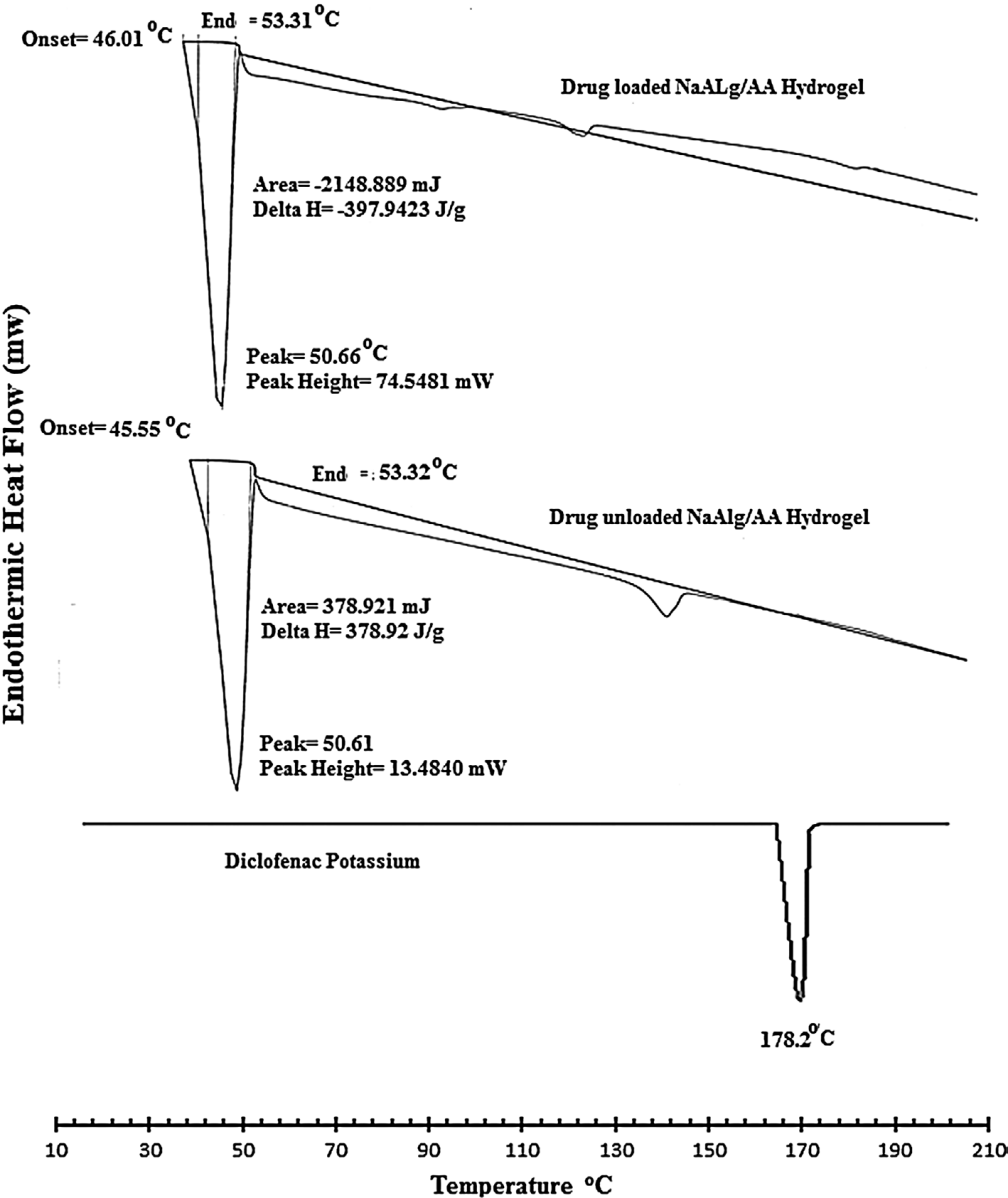
DSC spectra of pure drug (diclofenac potassium), loaded NaAlg/AA hydrogel and unloaded NaAlg/AA hydrogel.

DSC thermo grams of pure drug, unloaded, and drug-loaded hydrogels are presented in Figure 11. The thermo gram of DSC clearly indicates a sharp melting peak of diclofenac potassium at about 178.2 °C. The drug-loaded hydrogel showed an absence of drug melting peak which indicates molecular dispersion of drug in the prepared hydrogels. The unloaded sample did not show any endothermic transitions due to rigid polymer network structure because of chain entanglement.

### XRD analysis

**Figure 12.**
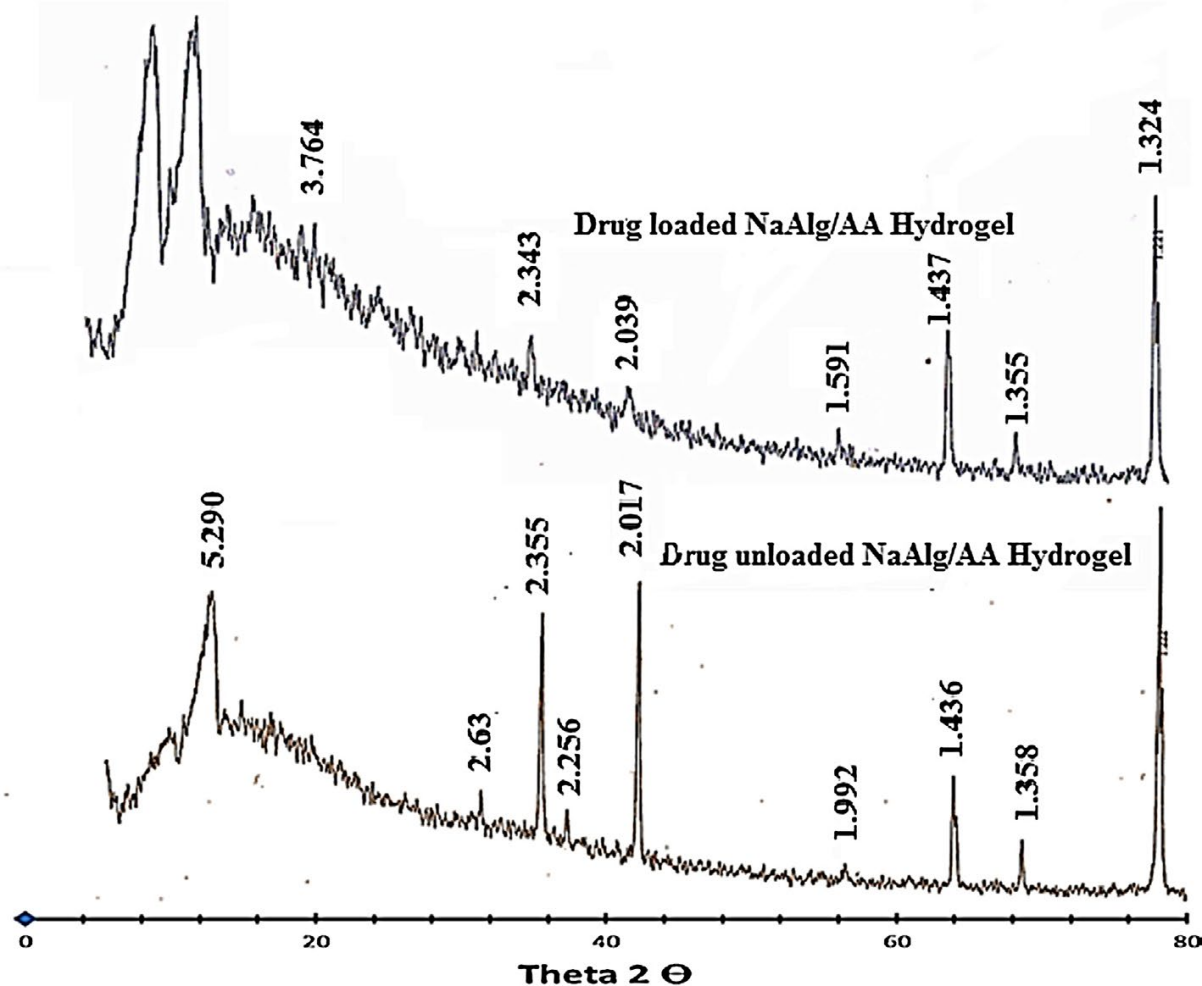
XRD pattern of loaded and unloaded NaAlg/AA hydrogel.

The XRD pattern of NaAlg/AA hydrogel and drug loaded NaAlg/AA hydrogel has been depicted in Figure 12. The diffract gram of unloaded NaAlg/AA hydrogel indicated peak at ~16.840°, 34.360°, 38.180°, 39.920°, 44.440°, 57.860°, 64.780°, 69.220°, 77.940°, 78.180° (2*θ*). While the diffract gram of drug loaded NaAlg/AA hydrogel indicated peaks at ~16.940°, 34.360°, 38.400°, 39.920°, 44.540°, 57.900°, 64.840°, 69.260°, 77.980°, 78.220° (2*θ*), which are nearly same as that of NaAlg/AA hydrogel sample without drug. It means that there is no apparent interaction reported between drug and hydrogel.

### Scanning electron microscopy

**Figure 13.**
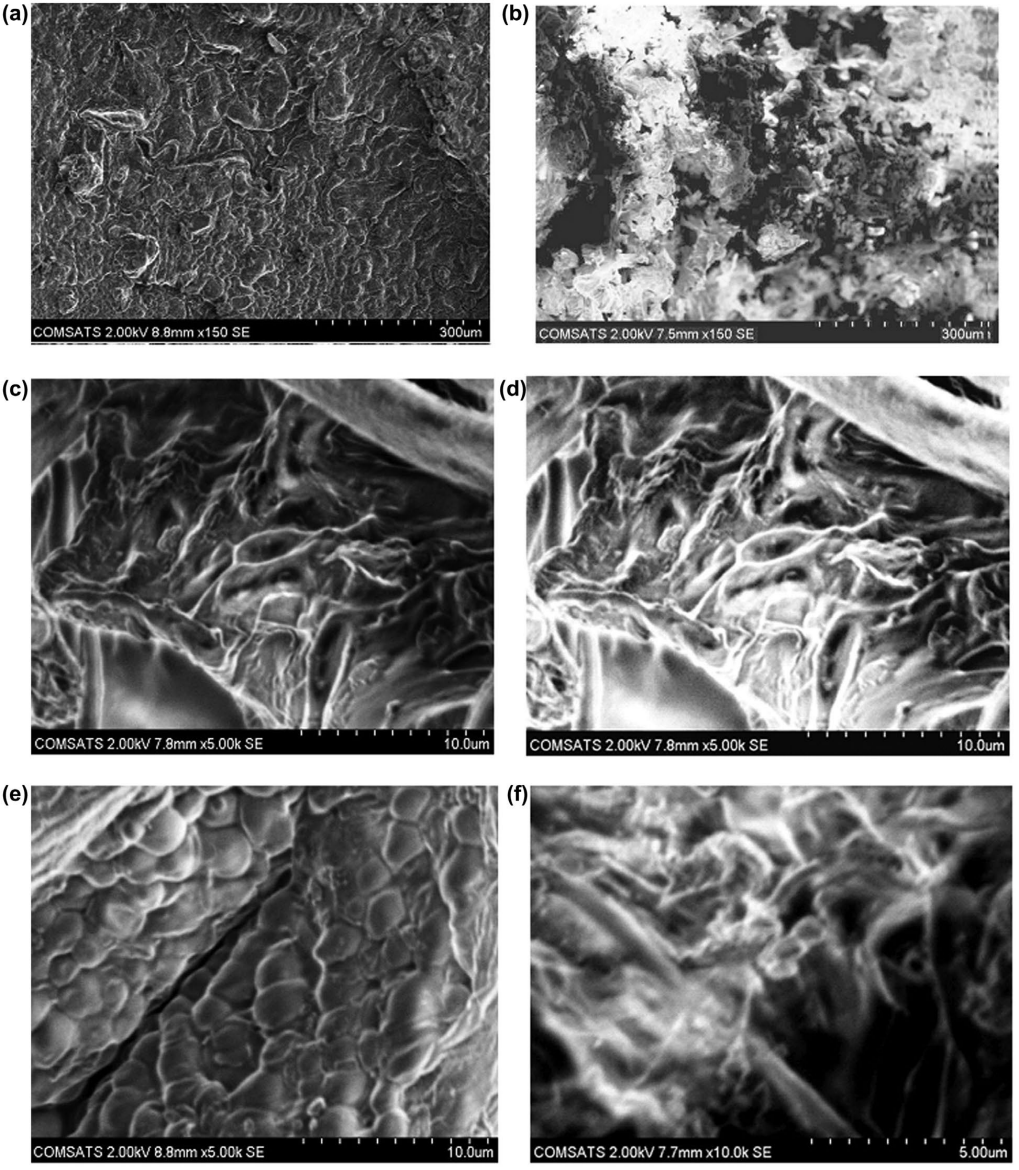
SEM micrographs of NaAlg/AA hydrogels (a) Surface morphology of unloaded hydrogel (b) Surface morphology of loaded hydrogel (c, e) Cross sectional morphology of unloaded hydrogel and (d, f) Cross sectional morphology of loaded hydrogel at various resolutions.

To study the surface morphology of the NaAlg/AA hydrogels, SEM of both loaded and unloaded samples were performed. Figure 13 elaborate the surface phenomena and cross section of the unloaded and loaded hydrogel samples. The results reveal the porous structure of the hydrogels which facilitate the adherence of the drug into the interpenetrating network of the hydrogel. This confirms the results obtained from porosity measurement of the hydrogels. Furthermore the uniform distribution of the drug within the matrix of the hydrogels in loaded sample is also confirmed.

## Conclusion

In present work pH sensitive highly porous cross-linked sodium alginate/acrylic acid (NaAlg/AA) hydrogels were prepared by free radical polymerization using ethylene glycol dimethacrylate (EGDMA) as crosslinking agent as a carrier for water soluble drugs. Composition of the hydrogel and pH of the medium decides the swelling behavior of the hydrogel. At different pH of buffer solution the swelling studies were carried out. The result demonstrated that with change of the composition of monomer, polymer and cross linker, the water uptake behavior of hydrogels changes in the buffer medium. The swelling ratio decreased with the increased concentration of monomer (AA) and cross linker (EGDMA) while increases with increasing concentration of polymer (NaAlg). The pH of the medium and composition of the hydrogel have also affected the drug release. In 0.05 M USP buffer of pH 7.5 the rate of drug release was faster as compared to other lower pH. It was concluded the NaAlg/AA hydrogel are pH sensitive and change their swelling and drug release behavior according to the surrounding medium. Therefore these polymeric formulations are suitable for controlled DDS. Drug release from all the formulations follow non-fickian pattern. FTIR spectroscopy was carried out for the structural confirmation of the hydrogel. The XRD study of the samples confirms the addition of diclofenac potassium. Thermal stability of the hydrogel samples was confirmed through DSC thermograms. SEM microscopy was carried out to investigate and confirm the porous network of NaAlg/AA hydrogel that facilitates the addition of model drug.

## Disclosure statement

No potential conflict of interest was reported by the authors.
